# Induction of p53-independent apoptosis by ectopic expression of HOXA5 in human liposarcomas

**DOI:** 10.1038/srep12580

**Published:** 2015-07-29

**Authors:** Dhong Hyun Lee, Charles Forscher, Dolores Di Vizio, H. Phillip Koeffler

**Affiliations:** 1 Division of Hematology and Oncology, Departments of Surgery,Biomedical Sciences and Pathology and Laboratory Medicine, Samuel Oschin Comprehensive Cancer Institute,Cedars-Sinai Medical Center, Los Angeles, CA, USA; 2 Division of Cancer Biology and Therapeutics, Departments of Surgery, Biomedical Sciences and Pathology and Laboratory Medicine, Samuel Oschin Comprehensive Cancer Institute, Cedars-Sinai Medical Center, Los Angeles, CA, USA; 3 The Urological Diseases Research Center; Boston Children’s Hospital, Boston, MA, Department of Surgery, Harvard Medical School, Boston, MA, USA; 4 National Cancer Institute and Cancer Science Institute, National University of Singapore, Singapore.

## Abstract

Dedifferentiated liposarcoma (DDLPS) is a highly malignant subtype of human liposarcoma (LPS), whose genomic profile is characterized by chromosomal amplification at 12q13-q22. miR-26a-2 is one of the most frequently amplified genes in the region, and inhibition of its downstream target genes likely contributes to LPS tumorigenesis. Our previous study of LPS predicted homeobox protein A5 (HOXA5) as a target of miR-26a-2, and here we explored further the function of HOXA5, and its relationship with miR-26a-2 in DDLPS cells. Compared to normal human adipocytes, all LPS cell lines showed significant downregulation of HOXA5 (p = 0.046), and inhibition of miR-26a-2 using anti-miR-26a-2 substantially upregulated HOXA5 expression in these LPS cells. Interestingly, overexpression of HOXA5 alone induced very strong apoptotic response of LPS cells. HOXA5-induced apoptosis was p53-independent and caspase-dependent. Surprisingly, overexpression of HOXA5 induced nuclear translocation of RELA (p65), which was not associated with the transcriptional activity of RELA. Rather, nucleolar sequestration of RELA was observed. Overall, our study demonstrated for the first time that the downregulation of HOXA5 in LPS cells, partly by overexpression of miR-26a-2 in DDLPS, confers LPS cells resistance to apoptotic death. Further studies are required to understand the relationship of HOXA5 and the NFκB pathway in LPS cells.

Human liposarcoma (LPS) is the most common soft-tissue sarcoma, and de-differentiated liposarcoma (DDLPS) is a highly malignant LPS subtype whose genomic profile is characterized by chromosomal amplification at 12q13-q22. These amplified region contains two distinct and independent amplicons in 90% of the cases, one centered at MDM2 and the other centered at miR-26a-2^1^.

The role of MDM2 in DDLPS tumorigenesis has been well studied. MDM2 is an E3 ubiquitin-protein ligase and an important inhibitor of p53 tumor-suppressor protein^2^. High MDM2 protein level in DDLPS cells keeps the endogenous p53 protein level low, and therefore provides resistance to p53-mediated apoptotic cell death. For this reason, Nutlins such as RG7112, the inhibitors of MDM2-p53 protein interaction, have been tested as potential chemotherapeutic agents for DDLPS. However, its clinical efficacy to date is disappointing^3^.

Unlike MDM2, the function of miR-26a-2 in DDLPS is only beginning to be understood. miR-26a-2 is a short, non-coding microRNA that can post-transcriptionally regulate multiple target genes in a cell-type specific manner. In our previous study, we found that overexpression of miR-26a-2 is strongly correlated with poor patient survival^1^. During the study, we identified 93 putative target genes of miR-26a-2, which could potentially impact LPS tumorigenesis in various ways. We studied one of the target genes, RCC1 and BTB domain-containing protein 1 (RCBTB1), and found that its inhibition by miR-26a-2 provided DDLPS cells resistance to apoptotic death^1^. To expand our understanding of miR-26a-2, we focused on HOXA5, another target gene of miR-26a-2 in LPS cells.

HOXA5 shows strong correlation to adipocyte differentiation and fat metabolism. HOXA5 is highly expressed in intra-abdominal adipocytes, and its expression level positively correlates with the extent of obesity and the pattern of fat distribution in both visceral and subcutaneous human white adipose tissues^4^,^5^,^6^. Therefore, aberrant HOXA5 expression can lead to various diseases, including cancer. In human breast cancer, loss of HOXA5 expression occurs, partly by methylation of the HOXA5 promoter^7^. Transcriptional upregulation of p53 and subsequent p53-dependent apoptosis resulted from the overexpression of HOXA5 in MCF7 cells^7^. HOXA5 was also able to induce apoptosis in a p53-independent manner in Hs578T cells which carries a mutant p53^8^.

Considering the oncogenic role of miR-26a-2 in human LPS cells, we hypothesized a tumor suppressive role of HOXA5 in DDLPS cells. Transcriptional inhibition of HOXA5 by miR-26a-2 provided resistance to apoptotic death in DDLPS cells. While exploring the molecular mechanism of HOXA5-induced apoptosis, we observed the potential involvement of the NFκB pathway, which may provide clues in understanding the role of HOXA5 in LPS tumorigenesis.

## Results

### Identification of HOXA5 as a target of miR-26a-2 in human LPS cells

**Figure 1:**
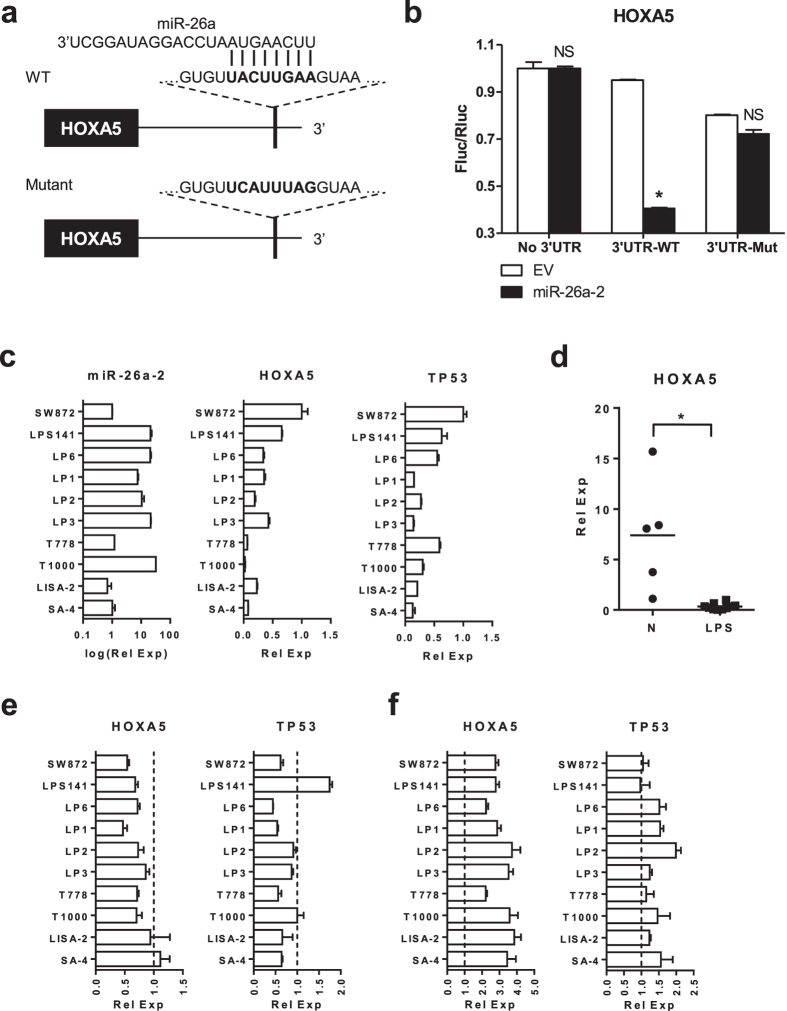
HOXA5 as a target of miR-26a-2 in LPS cells. (**a**) Schematic diagram of HOXA5 3′UTR showing seed sequence (UACUUGAA) of miR-26a-2 binding site. In mutant construct, the seed sequence was mutated to UCAUUUAG by site-directed mutagenesis. WT = wild type. (**b**) Summary of dual luciferase assays. pGL3-Promoter 3′UTR luciferase reporter vector was cotransfected with either miR-26a-2 expression vector or empty vector control (EV) in 293T cells. After 48 h, cells were harvested and luciferase activity was quantified. Assays were repeated three times to ensure accuracy. Fluc/Rluc = Firefly luciferase/Renilla luciferase. Data represent average Fluc/Rluc ratio ± standard deviation (SD, error bars). Asterisk (*) indicate p-value less than 0.05 by t-test. NS = not significant. (**c**) Endogenous mRNA expression level of miR-26a-2, HOXA5, and TP53 in LPS cell lines. Data represent average relative mRNA expression (Rel. Exp.) ± SD. (**d**) Endogenous mRNA expression level of HOXA5 in normal adipose tissue (N) and LPS cell lines (LPS) shown in panel c. Each dot represents HOXA5 expression level of each sample. Horizontal bars represent average HOXA5 expression level within the group. (**e**) Effect of forced expression of miR-26a-2 on the mRNA expression level of HOXA5 and TP53 in LPS cell lines. Cells were transfected with either miR-26a-2 expression vector or EV. After 48 h, cells were harvested and subjected to qRT-PCR. Dashed lines indicate the expression level of each gene in cells with EV. (**f**) Effect of inhibition of miR-26a-2 using anti-miR-26a-2 oligos on the mRNA expression level of HOXA5 and p53 in LPS cells. Cells were transfected with either scrambled oligos (SCR) or anti-miR-26a-2. After 48 h, cells were harvested and subjected to qRT-PCR. Dashed lines indicate the expression level of each gene in cells with SCR.

We first examined if HOXA5 mRNA is a direct target of miR-26a-2 as predicted in our previous study^1^. Dual luciferase reporter assay results confirmed that miR-26a-2 could bind to the putative binding site in the HOXA5 3′UTR and achieved a 60% knockdown of the luciferase activity (p = 0.023) (Fig. 1a,b). The luciferase activity was partially restored with a point mutation at the miR-26a-2 binding site, confirming that the binding was site-specific.

Next, correlation between the endogenous expression levels of miR-26a-2 and HOXA5 was examined in 10 human LPS cell lines of various subtypes (Supplementary Table S1). High expression levels of miR-26a-2 were observed in DDLPS cell lines (LPS141, LP6, LPS1, LPS2, LPS3, T1000) (Fig. 1c). Interestingly, very low expression levels of HOXA5 were observed in all LPS cell lines, regardless of their subtypes (Fig. 1c). Average expression level of HOXA5 in the 10 LPS cell lines was significantly lower than in normal human adipocyte tissues (p = 0.046) (Fig. 1d). Very low expression levels of TP53 were observed in all LPS cell lines, regardless of their subtypes (Fig. 1c).

To demonstrate that HOXA5 expression can be regulated by miR-26a-2 in LPS cells, we modulated miR-26a-2 expression and examined the changes in HOXA5 expression (Fig. 1e,f) (Supplementary Figure S1). Overexpression of miR-26a-2 significantly decreased the expression of HOXA5 in most of the LPS cell lines (p < 0.05 for all) except LISA-2 and SA-4 cell lines, and the inhibition of miR-26a-2 using anti-miR-26a-2 oligos produced the opposite results in all of the cell lines (p < 0.05 for all) (Fig. 1e,f). Modulation of HOXA5 expression by miR-26a-2 did not alter TP53 expression level in LPS cells (Fig. 1e,f). Overall, the results suggested that miR-26a-2 could regulate HOXA5 expression in LPS cell lines, particularly in the DDLPS subtype.

### Pro-apoptotic role of HOXA5 in human LPS cells

**Figure 2:**
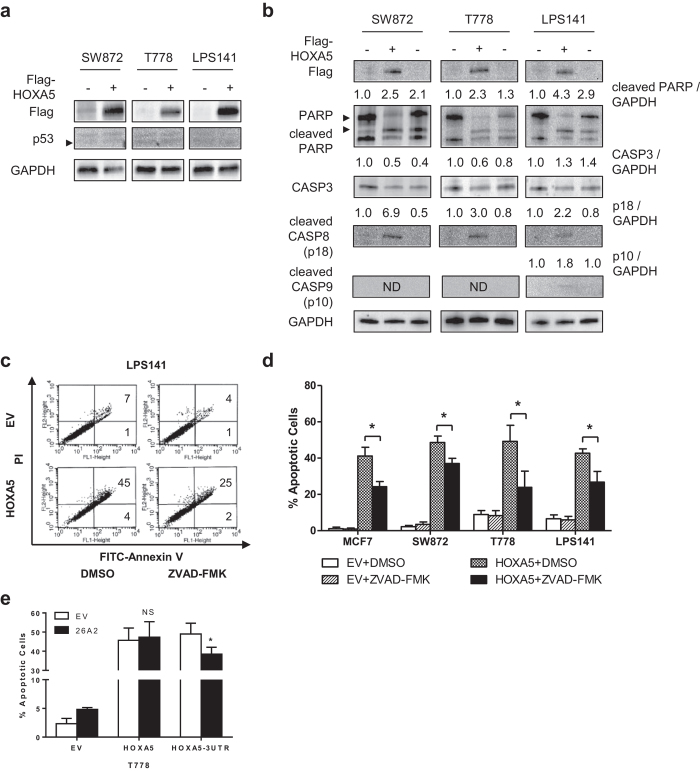
Forced expression of HOXA5 induces apoptosis of LPS cells. For panels a and b, LPS cells were transfected with either HOXA5 expression vector or empty vector control (EV). Cells were harvested 24 h after transfection and subjected to Western blot analyses. GAPDH was used as a loading control. (**a**) Representative Western blot images of p53. (**b**) Representative Western blot images of PARP and selected caspases. Third lane of each cell line is 5 µM doxorubicin-treated positive control. Numbers indicate relative band intensity of each gene normalized to the intensity of the genes in EV (1.0) for each fraction. For panels c and d, LPS cells were transfected with either HOXA5 expression vector or EV, and subsequently treated with 10 μM ZVAD-FMK 12 h after transfection. Cells were further incubated for an additional 12 h, and subjected to apoptosis assay. MCF7 cell line was used as a positive control. (**c**) Representative apoptosis assay results of LPS141 cells. Numbers indicate the percentage of early-apoptotic (bottom) and late-apoptotic (top) cells. (**d**) Summary of apoptosis assay results. Data represent % apoptotic cells ± standard deviation (SD, error bars). Asterisk (*) indicates p-value less than 0.05 by t-test. (**e**) Effect of miR-26a-2 on HOXA5-induced apoptosis. Cotransfection of miR-26a-2 with either HOXA5 (no 3′UTR) or HOXA5-3′UTR (having miR-26a-2 binding site) expression vector was done in T778 cells. Graph shows the summary of apoptosis assay results. NS = not significant.

Low endogenous expression level of HOXA5 and the downregulation of HOXA5 by oncogenic miR-26a-2 suggested that HOXA5 has a tumor-suppressive role in LPS cells. Due to its correlation to adipocyte differentiation and fat metabolism^4^,^5^,^6^, potential involvement of HOXA5 in regulating key adipocyte differentiation marker proteins in LPS cells was examined. For these and further studies, we selected SW872, T778, and LPS141 cell lines as representatives of undifferentiated, well-differentiated, and de-differentiated LPS subtypes, respectively. Overexpression of HOXA5 had little effect or even decreased the expression of key genes that mediate adipocyte differentiation (CBEPA, CEBPB, PPARG) in these LPS cells (Supplementary Figure S2). Rather, overexpression of HOXA5 induced a very strong apoptotic response as early as 24 hours after transfection (Fig. 2a–d). A similar apoptotic response upon HOXA5 overexpression has been reported in MCF7 cell line, which was used as a positive control (Fig. 2d)^7^. Few apoptotic cells were observed using empty vector control, confirming that the observed apoptosis was not due to non-specific cytotoxicity of the transfection. This apoptotic response was partially diminished when HOXA5 expression vector containing 3′UTR region of miR-26a binding site (HOXA5-3UTR) was cotransfected with miR-26a-2 expression vector (26A2) in T778 cells (p = 0.037) (Fig. 2e). This reduction of apoptosis was not observed with HOXA5 expression vector lacking its 3′UTR region. Overexpression of HOXA5 also induced cleavage of PARP protein as well as various caspases (Fig. 2b). Addition of ZVAD-FMK, a pan-caspase inhibitor, after HOXA5 transfection significantly reduced the number of apoptotic cells (p < 0.05 for all) (Fig. 2c,d). Possible induction of necroptosis by HOXA5 overexpression has also been tested. However, addition of necrostatin-1 (Nec-1), a necroptosis inhibitor, after HOXA5 transfection failed to reduce HOXA5-induced cell death (Supplementary Figure S3).

Interestingly, HOXA5 overexpression in SW872, T778, and LPS141 cells caused apoptosis irrespective of their MDM2 and p53 status (Supplementary Table S1), implying that p53, a major activator of apoptosis^9^, may not be involved in the process in these cells. Indeed, overexpression of HOXA5 did not upregulate the protein expression of p53 (Fig. 2a).

### Lack of correlation between HOXA5 and TP53 expression in human LPS cells

**Figure 3:**
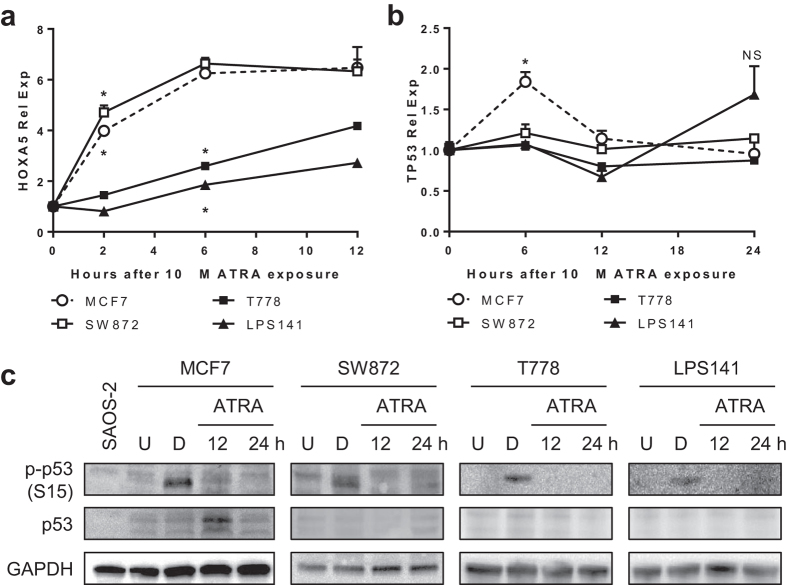
Effect of all-trans retinoic acid (ATRA) on the expression of HOXA5 and TP53 in LPS cells. LPS cells were treated with 10 μM ATRA for 2–12 h. At each time point, cells were harvested and subjected to qRT-PCR and Western blot analyses. MCF7 cell line was used as a positive control. (**a**,**b**) Changes in mRNA expression levels of HOXA5 (panel A) and TP53 (panel B) upon ATRA treatment. Data represent relative mRNA expression ± standard deviation (SD, error bars). GAPDH was used as a loading control. Asterisk (*) indicates p-value less than 0.05 by t-test. NS = not significant. (**c**) Representative Western blot images showing the changes of p53 protein levels in ATRA-treated LPS cells. SAOS-2 cell line is p53-negative and was used as a p53-negative control. For positive control of p53 activity upon cellular stress, cells were treated with 500 nM doxorubicin (D) for 6 h. U = Untreated control (DMSO-treated).

To rule out the possibility that ectopically expressed HOXA5 proteins in this experimental setting cannot induce TP53 expression, all-trans retinoic acid (ATRA)-based upregulation of endogenous HOXA5 gene was examined. ATRA is known to stimulate the expression of HOXA5 in MCF7 cells when ATRA-bound retinoic acid receptor β (RARB) binds to the retinoic acid response elements (RAREs) in the promoter region of HOXA5^10^. MCF7 cells (positive control) treated with 10 μM ATRA showed substantial increase in both HOXA5 and TP53 expression levels upon ATRA treatment (Fig. 3a,b). Although LPS cells at the same condition showed various levels of HOXA5 upregulation as early as 2 h after ATRA treatment, they did not show any increase in TP53 expression level (Fig. 3a,b). Western blot analysis further confirmed this idea. Whereas MCF7 cells showed transient increase in p53 protein level after 12 h of ATRA exposure, LPS cells at the same condition showed no such increase, even after 24 h of ATRA exposure (Fig. 3c). Burst of phospho-p53 (S15) upon 500 nM doxorubicin (D) treatment proved the presence of functional p53 in these LPS cells, ruling out the possibility that p53 proteins are absent in these cells (Fig. 3c).

### Nuclear localization of RELA protein upon HOXA5 expression in human LPS cells

Since p53 was not the activator of HOXA5-induced apoptosis in LPS cells, other apoptosis-related pathways were explored. Synergistic increase in apoptosis was reported in HOXA5-overexpressing Hs578T cells treated with TNF-α^8^. Although the molecular mechanism of synergism remained unclear, the result implied the potential interaction of HOXA5 and the NFκB pathway, considering TNF-α could mediate the degradation of NFKBIA (IκBα) and subsequent nuclear translocation of RELA (p65), a hallmark of activation of the NFκB pathway^11^. Activation of the NFκB pathway is generally considered anti-apoptotic and cytoprotective, but it can also be pro-apoptotic depending on the type of cellular stress^11^,^12^,^13^.

**Figure 4:**
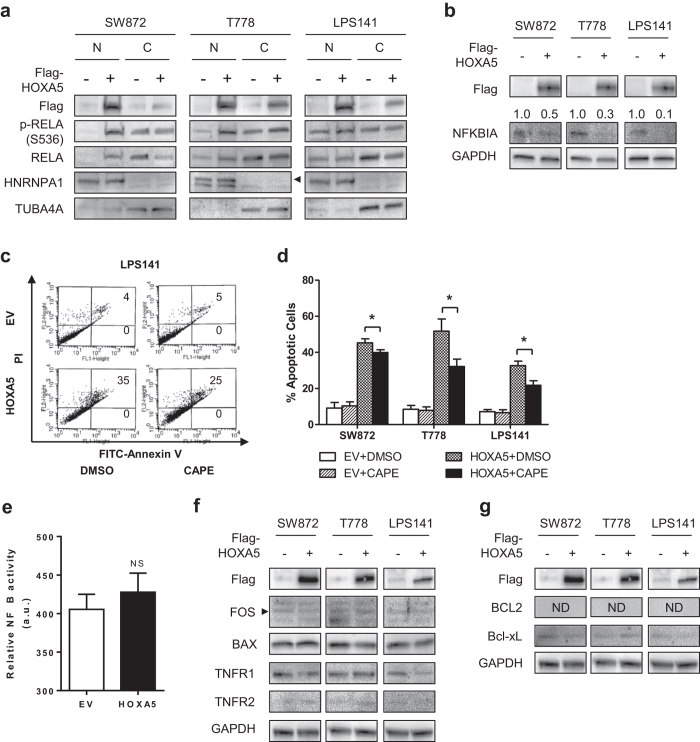
Effect of HOXA5 on the NFκB signaling pathway. For panels a and b, LPS cells were transfected with either HOXA5 expression vector or empty vector control (EV). Cells were harvested 12 h after transfection, and subjected to Western blotting. (**a**) Representative Western blot images showing subcellular distribution of RELA protein 12 h after transfection. HNRNPA1 and TUBA4A were used as loading controls for nuclear (N) and cytoplasmic (C) fraction of cells, respectively. (**b**) NFKBIA level from cells under the same condition. For panels c and d, LPS cells were transfected with either HOXA5 expression vector or EV, treated with 25 μg/ml CAPE 9 h after transfection, cultured for additional 15 h, and subjected to apoptosis assay. (**c**) Representative apoptosis assay of LPS141 cells. Numbers indicate the percentage of early-apoptotic (bottom) and late-apoptotic (top) cells. (**d**) Summary of apoptosis assay results. Data represent % apoptotic cells ± standard deviation (SD, error bars). Asterisk (*) indicates p-value less than 0.05 by t-test. (**e**) Summary of dual luciferase assay results. T778 cells were transfected with either 3×κB ConA luciferase reporter vector or ConA vector control without κB element. 24 h after transfection, cells were subsequently transfected with either HOXA5 expression vector (HOXA5) or empty vector control (EV). 12 h after transfection, cells were harvested and luciferase activity was quantified. Data represent average Fluc/Rluc ratio ± SD. a. u. = arbitrary unit, NS = not significant. For panels f and g, LPS cells were transfected with either HOXA5 expression vector or empty vector control (EV). Cells were harvested 24 h after transfection, and subjected to Western blotting. (**f**) Representative Western blot images showing protein levels of selected pro-apoptotic NFκB target proteins. (**g**) Representative Western blot images showing protein levels of selected anti-apoptotic NFκB target proteins.

Because significant number of cells underwent apoptotic response as early as 24 h after transfection in our experimental settings (Fig. 2), HOXA5 expression at an early time point was examined. Western blot analysis showed significant HOXA5 expression 12 h after its transfection, along with the nuclear translocation of the protein (Fig. 4a). At the same time, significant increase in nuclear translocation of RELA protein was observed in these LPS cells (Fig. 4a), as well as degradation of NFKBIA protein (Fig. 4b). When LPS cells were treated with 25 mg/ml caffeic acid phenethyl ester (CAPE), an inhibitor of RELA nuclear translocation (Supplementary Figure S4)^14^, significantly reduced apoptotic response was observed in the HOXA5-overexpressing LPS cells (p < 0.05 for all) (Fig. 4c,d).

Considering RELA is a transcription factor, NFκB transcriptional activity was examined in HOXA5-overexpressing T778 cells using 3×κB ConA luciferase reporter vector. However, no significant change in NFκB transcriptional activity upon HOXA5 overexpression was observed (p = 0.291) (Fig. 4e). Expression levels of several known NFκB target genes related to apoptosis were also examined. Neither key pro-apoptotic (FOS, BAX, TNFR1, TNFR2, FAS, FASL) nor anti-apoptotic (BCL2, Bcl-xL, BAD) NFκB target genes showed any significant change in their expression levels upon HOXA5 overexpression in LPS cells (Fig. 4f,g) (Supplementary Figure S5). Therefore, nuclear translocation of RELA in HOXA5-overexpressing LPS cells was independent of NFκB transcriptional activity.

**Figure 5:**
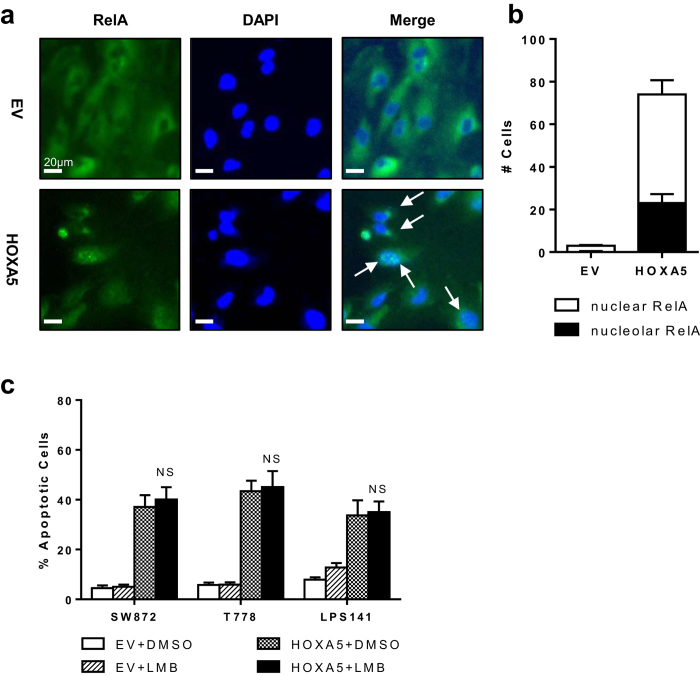
Effect of forced expression of HOXA5 on subcellular distributions of RELA in LPS cells. For panels a and b, T778 cells were transfected with either HOXA5 expression vector or empty vector control (EV), and subjected to immunocytochemistry with RELA antibody 12 h after transfection. (**a**) Representative immunofluorescent images are shown. Nuclei were stained with DAPI. Arrows indicate the location of RELA puncta. ×40. Size bar = 20 μm. (**b**) Cell counting summary showing number of cells with nuclear RELA and nucleolar RELA (presence of RELA puncta). Data represent average number of cells in a given field ± standard deviation (SD, error bars). (**c**) Summary of apoptosis assay results. LPS cells were transfected with either HOXA5 expression vector or EV. Cells were treated with 100 nM leptomycin B (LMB) 9 h after transfection, and incubated further for an additional 15 h. Data represent % apoptotic cells ± SD. NS = not significant.

Recent report showed that nucleolar sequestration of RELA could be pro-apoptotic, independent of RELA transcriptional activity, by causing cytoplasmic translocation of nucleophosmin (NPM)^12^. We used immunocytochemistry (ICC) to check the localization of the RELA protein in T778 cells. In empty-vector transfected control (EV), RELA was predominantly localized in the cytoplasm (Fig. 5a,b). In HOXA5 overexpressing cells (HOXA5), the fraction of nuclear RELA greatly increased, and high-density RELA puncta (indicated by arrows) was observed within the nuclei of the cells, consistent with nucleolar sequestration of RELA (Fig. 5a,b). To examine if nuclear export of NPM played a role, T778 cells were treated with leptomycin B (LMB), an inhibitor of nuclear export pathway called RAN-exportin-1 (CRM1p) pathway^12^. However, 20 nM LMB failed to block HOXA5-induced apoptosis of T778 cells, and higher dose of LMB (100 nM) did not change the results (Fig. 5c). Overall, these experimental results confirmed that nucleolar sequestration of RELA might be one of the likely mechanisms of HOXA5-induced apoptosis in LPS cells.

## Discussion

HOX genes are a group of transcription factors containing a DNA binding domain called homeobox domain. These highly conserved homologues are located contiguously in clusters in vertebrate genomes, and their spatiotemporal expression is tightly regulated and balanced throughout embryonic development and normal organogenesis^15^. HOX genes are known to regulate various cellular processes including cell proliferation and survival, differentiation, apoptosis, cell motility, epithelial-mesenchymal transition (EMT), and receptor signaling^15^. For these reasons, deregulation of HOX genes can result in various developmental defects and other diseases, including cancer.

Recently, an increasing number of studies have reported aberrant expression of HOX genes in cancer. Interestingly, the role of HOX genes in cancer is cell type-specific. For example, HOXB13 have tumor suppressive effects in prostate and lung cancers, and oncogenic effects in ovarian and breast cancers^15^. Likewise, level of HOXA5 protein varies depending on the cancer type. According to Oncomine (http://www.oncomine.org)^16^, HOXA5 is highly expressed in AML, gastrointestinal stromal tumors, and glioblastomas, whereas it is weakly expressed in breast, colon, lung, and ovarian cancers. Interestingly, modest upregulation of HOXA5 was noted in myxoid LPS (MRC) (p = 2.20E-8, fold change = 5.861). Due to the unavailability of LPS cell lines with MRC subtype, we could not confirm this finding in our study. It would be interesting to explore the differential function of HOXA5 in different LPS subtypes.

We found that HOXA5 expression could be regulated at the post-transcriptional level by miR-26a-2 in LPS cells. In DDLPS, high expression of miR-26a-2 due to copy number gain kept HOXA5 expression level low. Similarly, HOXA5 is also regulated by miR-130 in human breast cancer cells^17^. In breast cancer cells, high expression of c-myc and subsequent high expression of miR-130a resulted in low HOXA5 expression^17^. Due to the lack of high levels of either c-myc or miR-130a^1^, miR-130a is unlikely to play a role in the regulation of HOXA5 in LPS cells.

We selected SW872, T778, and LPS141 cell lines as representatives of undifferentiated, well-differentiated, and de-differentiated LPS subtypes, respectively. All three cell lines showed similar apoptotic response to HOXA5 expression at the cellular level, but some differences existed at the molecular level. For example, SW872 and T778 showed caspases cleavage (Fig. 2b). LPS141 demonstrated cleavage of CASP8 and CASP9, but not CASP3. Understanding these differences may help to determine the molecular mechanism of HOXA5-induced apoptosis, as well as to understand the molecular differences of these LPS subtypes.

We initially hypothesized that increased TP53 expression by HOXA5 could explain HOXA5-induced apoptosis. However, neither ectopic expression of HOXA5 nor the induction of endogenous HOXA5 by ATRA could induce TP53 expression in LPS cells (Figs 2,3). Due to the HOXA5 binding site in the TP53 promoter^7^, epigenetic silencing of TP53 by methylation of CpG islands around the TP53 transcription start site was speculated. However, TP53 methylation is extremely rare event in soft tissue sarcoma^18^, and is unlikely to explain the unresponsiveness of TP53.

Activation of the NFκB pathway may explain HOXA5-induced apoptosis. How HOXA5 induces nuclear localization of RELA protein is unclear. Depletion of RELA protein using shRNA was cytotoxic to LPS cells (Supplementary Figure S6), and LPS cells seem to require threshold RELA activity for their survival. Considering rapid nuclear translocation of RELA upon HOXA5 expression, transcriptional activity of HOXA5 is unlikely to be associated. Direct protein-protein interaction or cofactor-mediated interaction between HOXA5 and RELA may explain this phenomena. It may imitate the predicted protein-protein interaction between HOXB7 and NFKBIA through ankyrin repeat domain^19^.

In terms of apoptosis, nuclear localization of RELA can lead to three possible consequences. In general, nuclear translocation of RELA leads to the transcriptional activation of the NFκB pathway. Therefore, transcriptional upregulation of pro-apoptotic target genes is conceivable^11^. On the contrary, nuclear translocation of RELA can also lead to downregulation of NFκB transcriptional activity, which results in the downregulation of anti-apoptotic target genes such as BCL2^20^. Alternatively, nucleolar sequestration of RELA can lead to apoptosis independent of its transcriptional activity^12^. HOXA5-induced apoptosis seems to follow the transcription-independent mechanism. However, it was not dependent on the cytoplasmic translocation of NPM, and the molecular mechanism of apoptosis remains unclear.

HOXA5-mediated apoptosis could be utilized for the development of potential therapeutic approaches for DDLPS treatment. Induction of HOXA5 using ATRA as a therapeutic option was considered but discarded due to the high resistance of LPS cells to ATRA (IC_50_ > 50 μM for all). Induction of HOXA5 using anti-miR-26a-2 oligos could be another possible therapeutic option which requires further studies.

In conclusion, this study identified for the first time that HOXA5 is a direct target of miR-26a-2. Furthermore, low HOXA5 expression level mediated by high expression of miR-26a-2 in DDLPS cells confers resistance to apoptotic death. We also identified that HOXA5-induced apoptosis in LPS cells was p53-independent and caspase-dependent, regardless of LPS subtypes or p53 status of the cells. In addition, we identified that nuclear translocation of RELA played important roles in HOXA5-induced apoptosis in LPS cells, and nucleolar sequestration of RELA might be one of the likely mechanisms of apoptosis. In conclusion, our study showed the importance of downregulation of HOXA5 in LPS tumorigenesis.

## Methods

### Human LPS cell lines

Ten human LPS cell lines were used in the study. SW872 cells were purchased from American Tissue Type Culture Collection (ATCC, Rockville, MD). Information for other non-ATCC LPS cell lines are provided in Supplementary Table S1. 293T, MCF7, and SAOS-2 cell lines were used for functional studies. All ATCC cell lines (SW872, 293T, MCF7, SAOS-2) were authenticated by short tandem repeat (STR) profiling, and all DNA profiles for the cell lines matched the DNA profiles provided by DSMZ. All LPS cell lines were maintained in RPMI medium (Mediatech, Inc., Herndon, VA) supplemented with 15% fetal bovine serum (FBS) (Omega Scientific, Tarzana, CA) in a humidified incubator at 37 °C supplied with 5% CO_2_. 293T, MCF7, and SAOS-2 cell lines were maintained in DMEM medium (Mediatech, Inc.) supplemented with 10% FBS. Only cells in the exponential growth phase were used in the study.

### Chemical compounds

ZVAD-FMK was purchased from Santa Cruz Biotechnology (Dallas, TX). All-trans-retinoic acid (ATRA) and doxorubicin were obtained from Sigma-Aldrich (St. Louis, MO). Caffeic acid phenethyl ester (CAPE) was obtained from Selleck Chemicals (Houston, TX). Leptomycin b (LMB) and necrostatin-1 (Nec-1) was purchased from Cayman Chemicals (Ann Arbor, MI). ZVAD-FMK, ATRA, and Nec-1 were solubilized in DMSO to a stock concentration of 10 mM. CAPE was solubilized in DMSO to a stock concentration of 1 mg/ml. LMB was supplied at a stock concentration of 0.1 μg/μl in ethanol.

### Quantitative reverse-transcription real-time PCR (qRT-PCR)

RNA purification, cDNA construction, and qRT-PCR were performed as previously described^1^. Primer sets for the detection of genes of interest are listed in Supplementary Table S2.

### Western blot analysis

Western blotting was performed as previously described^1^. Antibodies used for Western blotting are listed in Supplementary Table S3. ImageJ was used to process blot images and analyze band intensities^21^.

### Apoptosis analysis

Apoptosis analysis was performed using either BD Pharmigen FITC Annexin V Apoptosis Detection Kit (BD Biosciences, San Jose, CA), or eBioscience Annexin V Apoptosis Detection Kit APC (eBioscience, San Diego, CA) as previously described^1^.

### Modulation of miR-26a-2 expression level in LPS cell lines

Modulation of miR-26a-2 expression level was performed as previously described^1^. Briefly, either pMSCV-miR-26a-2 or pcDNA3.1-miR-26a-2 expression vectors were used for the overexpression studies, and miScript anti-hsa-miR-26a-2*miRNA inhibitor oligos (Qiagen, Valencia, CA) were used for the inhibition studies. Modulation of miR-26a-2 expression levels in both studies were validated by qRT-PCR (Supplementary Figure S1).

### Modulation of HOXA5 expression level in LPS cell lines

For overexpression of HOXA5, pcDNA3.1-Flag-HOXA5 expression vector was constructed. Full-length cDNA sequence of human HOXA5 was PCR-amplified using HOXA5 CDS primer set (Supplementary Table S2), and transferred to pcDNA3.1-Flag vector using EcoRI restriction sites. LPS cells were transfected with either HOXA5 expression vector or empty vector control using jetPRIME DNA transfection reagent according to the manufacturer’s protocol (BIOPARC, Illkirch, France). Expression of HOXA5 was validated by qRT-PCR and Western blot.

### Modulation of RelA expression level in LPS cell lines

Two pLKO.1-shRelA vectors were kindly provided by Dr. Michael Cleary at Stanford University (Stanford, CA)^22^. pLKO.1-scrambled shRNA negative control vector (Addgene plasmid 1864) was obtained from Addgene (Cambridge, MA)^23^. Lentiviral packaging was performed in 293T cells as previously described^1^. LPS cell lines were transduced with viral supernatants of either shRelA vector or scrambled shRNA vector control with 4 μg/ml polybrene solution. After 48 h, stable clones were selected using puromycin. Inhibition of HOXA5 was validated by qRT-PCR and Western blot.

### Luciferase reporter assays

pGL3-Promoter vector containing the miR-26a-2 binding site in the 3′UTR region of the HOXA5 gene was constructed using primer sets shown in Supplementary Table S2. To introduce point mutations to the miR-26a-2 binding site, Quikchange II XL site-directed mutagenesis kit was used according to the manufacturer’s protocol (Stratagene, La Jolla, CA). Cotransfection of pGL3-Promoter-HOXA5 3′UTR vector and pRL-TK renilla luciferase vector, along with either miR-26a-2 expression vector or empty vector control, in 293T cells was performed using jetPRIME DNA transfection reagent according to the manufacturer’s protocol. After 48 h, cells were harvested and subjected to dual luciferase assay using Promega dual luciferase reporter assay kit according to the manufacturer’s protocol (Madison, WI).

The three enhancer CONA (3×κB ConA-Luc) luciferase reporter vector and the empty vector control (ConA-Luc) were kindly provided by Dr. R. T. Hay (University of Dundee, UK)^24^. Cotransfection of ConA vectors and pRL-TK renilla luciferase vector, along with either HOXA5 expression vector or empty vector control, was performed as described above.

### Immunocytochemistry (ICC)

Immunofluorescent staining of RelA in T778 cells was performed as previously described^12^. Briefly, T778 cells were seeded on sterilized cover glass and transfected with either HOXA5 expression vector or empty vector control as described above. 12 h after transfection, cells were fixed in 1:1 methanol:acetone for 20 min at −20 °C, and permeabilized in PBS with 0.1% Triton-X for 15 min at room temperature. Cells were blocked in blocking buffer (PBST with 10% goat serum), and incubated with primary and secondary antibodies using standard procedure. Antibodies used for ICC are listed in Supplementary Table S3. Coverslips were mounted in Vectashield mounting medium containing DAPI (4′,6′-diamido-2-phenylindole) (Vector Laboratories, Burlingame, CA), and images were taken using fluorescent microscopy. ImageJ was used to process the images^21^.

### Statistical Analysis

For all statistical tests, p-values less than or equal to 0.05 were considered statistically significant and marked by a single asterisk (*). P-value less than or equal to 0.001 were marked by double asterisks (**). Two-tailed student t-test with unequal variances was used to compare the difference between two experimental groups. All experiments were repeated at least three times to ensure accuracy. Pearson’s correlation test was used to measure the correlation between two groups. Correlation coefficient of 0.5 or higher was considered significant at the 0.05 level.

## Additional Information

**How to cite this article**: Lee, D.H. *et al.* Induction of p53-independent apoptosis by ectopic expression of HOXA5 in human liposarcomas. *Sci. Rep.* **5**, 12580; doi: 10.1038/srep12580 (2015).

## Supplementary Material

Supplementary Information
