# Supplementary material for: Induction of p53-independent apoptosis by ectopic expression of HOXA5 in human liposarcomas
Source: Sci Rep. 2015 Jul 29;5:12580. doi: 10.1038/srep12580 (PMC4518222; doi:10.1038/srep12580)
Supplement: Supplementary Information [file srep12580-s1.pdf]

Supplementary Information

Induction of p53-independent apoptosis by ectopic expression of HOXA5 in human liposarcomas

Dhong Hyun Lee[1, *], Charles Forscher[1], Dolores Di Vizio[2, 3], and H. Phillip Koeffler[1, 4].

[1]Division of Hematology and Oncology, [2]Division of Cancer Biology and Therapeutics, Departments of Surgery, Biomedical Sciences and Pathology and Laboratory Medicine, Samuel Oschin Comprehensive Cancer Institute, Cedars-Sinai Medical Center, Los Angeles, CA. [3]The Urological Diseases Research Center; Boston Children's Hospital, Boston, MA, Department of Surgery, Harvard Medical School, Boston, MA. [4]National Cancer Institute and Cancer Science Institute, National University of Singapore, Singapore.

*** Correspondence:**

Dhong Hyun Lee, Ph. D.

Division of Hematology and Oncology, Cedars-Sinai Medical Center, UCLA School of Medicine, 8700 Beverly Blvd, D5022, Los Angeles, CA 90048, USA; Phone: +1-310-423-7736; Fax: +1-310-423-0225; E-mail: hiromasa@ucla.edu

Supplementary Figure S1

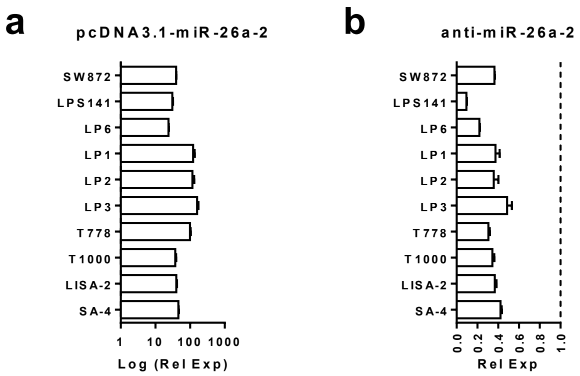

Supplementary Figure S1. Validation of miR-26a-2 modulation in LPS cells. (a) For the overexpression study shown in Figure 1e, LPS cells were transfected with either miR-26a-2 expression vector or empty vector control (EV). After 48 h of transfection, cells were harvested and subjected to qRT-PCR. Data represent average relative miR-26a-2 expression ± standard deviation (SD, error bars). Dashed line indicates the expression level of miR-26a-2 in cells with EV. (b) For the inhibition study shown in Figure 1f, LPS cells were transfected with either anti-miR26a-2 or scrambled oligos (SCR). After 48 h, cells were harvested and subjected to qRT-PCR. Dashed line indicates the expression level of miR-26a-2 in cells with SCR.

Supplementary Figure S2

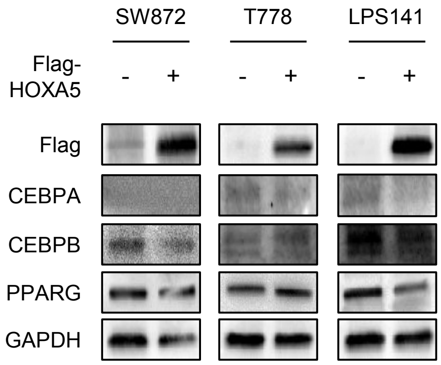

Supplementary Figure S2. Changes in the protein levels of key adipocyte differentiation proteins upon HOXA5 overexpression in LPS cells. LPS cells were transfected with either HOXA5 expression vector or empty vector control. Cells were harvested 24 h after transfection and subjected to Western blot analysis. Representative images are shown. GAPDH was used as a loading control.

Supplementary Figure S3

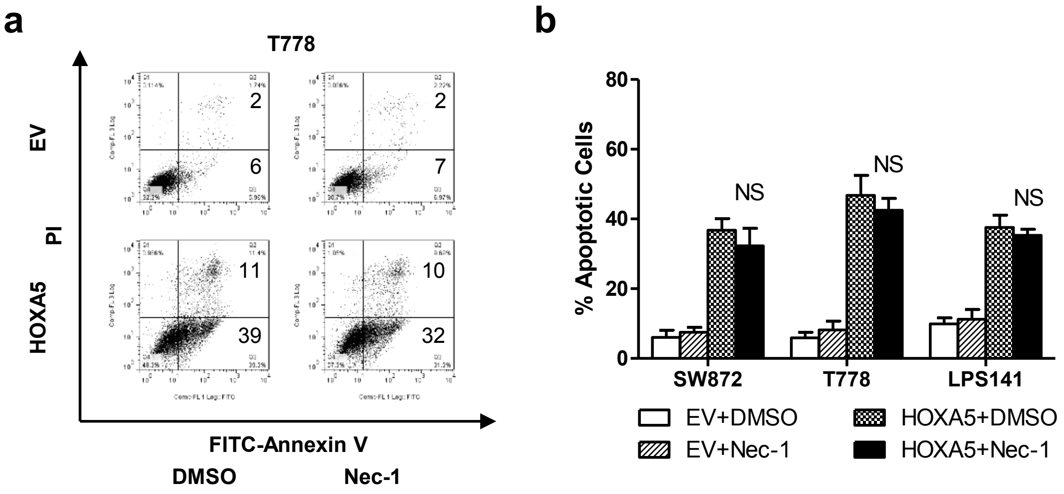

Supplementary Figure S3. Effect of necrostatin-1 (Nec-1) on the HOXA5-induced apoptosis in LPS cells. LPS cells were transfected with either HOXA5 expression vector or empty vector control (EV), and subsequently treated with 5 μM Nec-1 12h after transfection. Cells were further incubated for an additional 12h, and subjected to apoptosis assay. (a) Representative apoptosis assay results of T778 cells. Numbers indicate the percentage of early-apoptotic (bottom) and late-apoptotic (top) cells. (b) Summary of apoptosis assay results. Data represent % apoptotic cells ± standard deviation (SD, error bars). NS = not significant.

Supplementary Figure S4

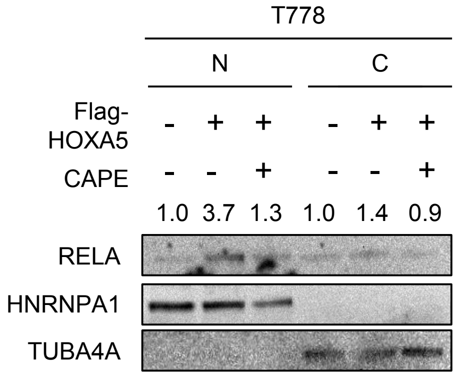

Supplementary Figure S4. Validation of caffeic acid phenethyl ether (CAPE) activity in LPS cells. T778 cells were transfected with either HOXA5 expression vector or empty vector control. Cells were treated with 25 µg/ml CAPE 9 h after transfection, incubated for an additional 15 h, and subjected to Western blotting. Representative images are shown. HNRNPA1 and TUBA4A (α-tubulin) were used as loading control for nuclear (N) and cytoplasmic (C) fraction of cells, respectively. Numbers indicate relative band intensity of RELA normalized to the intensity of the genes in control (1.0) for each fraction.

Supplementary Figure S5

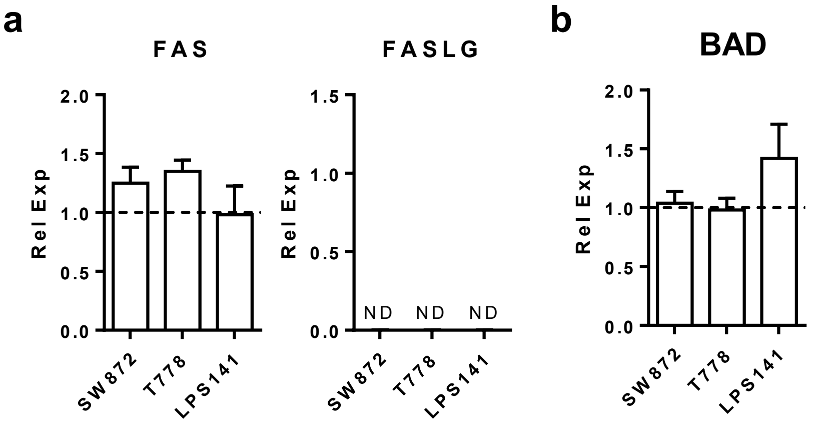

Supplementary Figure S5. Effect of HOXA5 overexpression on the transcription of selected NFκB target genes in LPS cells. SW872, T778, LPS141 cells were transfected with either HOXA5 expression vector or empty vector control (EV). 24h after transfection, cells were harvested and subjected to qRT-PCR (same condition as shown in Figures 4f and 4g). Primer pairs are shown in Supplementary Table S2. Graphs show changes in the mRNA expression level of selected pro-apoptotic (panel A) and anti-apoptotic (panel B) NFκB target genes upon HOXA5 overexpression in LPS cells. Dashed line indicates the expression level of each gene in cells with EV. Data represent relative mRNA expression ± standard deviation (SD, error bars). ND = no detection.

Supplementary Figure S6

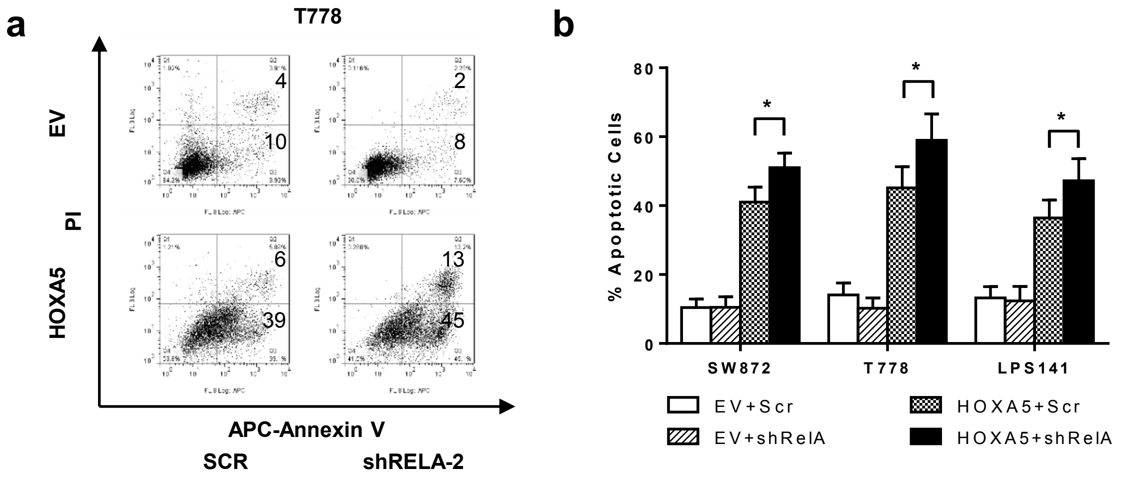

Supplementary Figure S6. Effect of RELA inhibition on the HOXA5-induced apoptosis in T778 cells. T778 cells stably transfected with either shRNA vector against RELA (shRELA-2) or scrambled control (SCR), and were subsequently transfected with either HOXA5 expression vector (HOXA5) or empty vector control (EV). Cells were incubated 24 h and subjected to apoptosis assay analysis. The other shRELA vector (shRELA-1) was cytotoxic and was excluded from the study. (A) Representative apoptosis assay results. Numbers indicate the percentage of early-apoptotic (bottom) and late-apoptotic (top) cells. (B) Summary of apoptosis assay results. Data represent % apoptotic cells ± standard deviation (SD, error bars). Asterisk (*) indicates p-value less than 0.05 by t-test.

Supplementary Table S1. Human liposarcoma (LPS) cell lines used in the study

Name	LPS subtype	Source	TP53 status	MDM2 status
SW872	Undifferentiated	ATCC[c]	Mutant<br>T356A (pI119N)	WT
LPS141	DDLPS[a]	Dr. Fletcher[d]	WT[h]	Overexpressed
LPS6	DDLPS	Dr. Fletcher	WT	Overexpressed
LPS1	DDLPS	Dr. Wu[e]	WT	Overexpressed
LPS2	DDLPS	Dr. Wu	WT	Overexpressed
LPS3	DDLPS	Dr. Wu	WT	Overexpressed
T778	WDLPS[b]	Dr. Pedeutour[f]	WT	Overexpressed
T1000	DDLPS	Dr. Pedeutour	WT	Overexpressed
LISA-2	Poorly differentiated	Dr. Möller[g]	Mutant<br>T926C (pL309S)	N/A
SA-4	N/A	N/A	WT	N/A

Note: [a]DDLPS = Dedifferentiated LPS; [b]WDLPS = Well-differentiated LPS; [c]ATCC = American Type Culture Collection; [d]Dr. Christopher D. M. Fletcher at Brigham and Women’s Hospital (Boston, MA)[1]; [e]Dr. Hong Wu at University of California, Los Angeles (Los Angeles, CA)[2]; [f]Dr. Florence Pedeutour at Nice University (Nice, France)[3]; [g]Dr. Peter Möller at University of Ulm (Ulm, Germany)[4]. [h]WT = Wild Type.

Supplementary Table S2. Primer sets used in this study

Gene ID	Forward Primer Sequence<br>Reverse Primer Sequence	Note
For quantitative reverse-transcription real-time PCR (qRT-PCR)		
GAPDH	5′-CAGCAAGAGCACAAGAGGAA-3′<br>5′-TCTACATGGCAACTGTGAGGAG-3′	Loading control
HOXA5	5′-CCAGATCTACCCCTGGATG-3′<br>5′-ACTTCATTCTCCGGTTTTGG-3′	
TP53	5′-CCCAAGCAATGGATGATTTGA-3′<br>5′-GGCATTCTGGGAGCTTCATCT-3′	
FASLG	5′-TCTACCAGCCAGATGCACAC-3′<br>5′-TCACTCCAGAAAGCAGGACA-3′	
FAS	5′-TCAGTACGGAGTTGGGGAAG-3′<br>5′-CCAATCCCTTGGAGTTGATG-3′	
BAD	5′-GCTGACCCAGATTCCCTTC-3′<br>5′-TAAACCTGGCTCGCGACTT-3′	
For cloning		
HOXA5<br>3’UTR-WT[a]	5′-GCCGTGTAATTCTAGAGTTCTCGTTGCCCTAA<br>TTCATC-3′<br>5′-CCGCCCCGACTCTAGAAGGAACACTTCCACGC<br>ACA-3′	pGL3
HOXA5<br>3’-UTR-Mut[b]	5'-AATAGATGTTTTAACTTATTTATATGAAGCAA<br>GCTGTGTTCATTTAGGTAACTATAACAAAAAAAGAAA<br>AGAGAAAAAAAAACACAC-3'<br>5'-GTGTGTTTTTTTTTCTCTTTTCTTTTTTTGTT<br>ATAGTTACCTAAATGAACACAGCTTGCTTCATATAAA<br>TAAGTTAAAACATCTATT-3'	pGL3
HOXA5<br>CDS[c]	5′-CGGAATTCATGAGCTCTTATTTTGTAAACTC-3′<br>5′-CGGAATTCTCAGGGACGGAAGGCCCCTCCT-3′	pcDNA3.1-Flag

Note: [a]WT = wild type; [b]Seed sequence of miR-26a-2 binding site (UACUUGAA) was mutated to UCAUUUAG by site-directed mutagenesis; [c]CDS = coding sequence

Supplementary Table S3. Antibodies used in this study

Antibody	Phosphorylation Site	Company	Catalog No
For Western blotting			
GAPDH		Santa Cruz	sc-47724
Flag		Sigma	F3165
phospho-p53	Ser15	Cell Signaling	9284
p53		Cell Signaling	2524
PARP		Cell Signaling	9532
CASP3		Cell Signaling	9668
CASP8		Santa Cruz	sc-7890
CASP9		Santa Cruz	sc-7885
phospho-RELA	Ser536	Cell Signaling	3033
RELA		Santa Cruz	sc-372
NFKBIA (IκBα)		Santa Cruz	sc-847
HNRNPA1		Santa Cruz	sc-10030
TUBA4A (α-tubulin)		Santa Cruz	sc-5286
FOS		Santa Cruz	sc-52
TNFR1		R & D Systems	MAB225
TNFR2		Santa Cruz	sc-8041
BAX		Santa Cruz	sc-493
BCL2		Santa Cruz	7382
Bcl-xL		Cell Signaling	2762
CEBPA		Santa Cruz	sc-9315
CEBPB		Santa Cruz	sc-150
PPARG		Cell Signaling	2243
For Immunocytochemistry			
RELA		Cell Signaling	6956
FITC-IgG		Abnova	PAB4971

REFERENCES TO SUPPLEMENTARY INFORMATION

1 Snyder, E. L. *et al.* c-Jun amplification and overexpression are oncogenic in liposarcoma but not always sufficient to inhibit the adipocytic differentiation programme. *J Pathol* **218**, 292-300, doi:10.1002/path.2564 (2009).

2 Smith, K. B. *et al.* Novel dedifferentiated liposarcoma xenograft models reveal PTEN down-regulation as a malignant signature and response to PI3K pathway inhibition. *Am J Pathol* **182**, 1400-1411, doi:10.1016/j.ajpath.2013.01.002 (2013).

3 Pedeutour, F. *et al.* Structure of the supernumerary ring and giant rod chromosomes in adipose tissue tumors. *Genes Chromosomes Cancer* **24**, 30-41 (1999).

4 Wabitsch, M. *et al.* LiSa-2, a novel human liposarcoma cell line with a high capacity for terminal adipose differentiation. *Int J Cancer* **88**, 889-894 (2000).
